# Household Vulnerability to Food Insecurity in Rural South Africa: Evidence from a Nationally Representative Survey Data

**DOI:** 10.3390/ijerph18041917

**Published:** 2021-02-17

**Authors:** Sandile Mthethwa, Edilegnaw Wale

**Affiliations:** 1 Inclusive Economic Development, Human Sciences Research Council, Berea, Durban 4001, South Africa; sandilemth@gmail.com; 2 Discipline of Agricultural Economics, University of KwaZulu-Natal, Pietermaritzburg 3209, South Africa

**Keywords:** vulnerability to food insecurity, VEP model, sustainable livelihoods framework, rural households, South Africa

## Abstract

Using a nationally representative dataset from rural areas in South Africa, the study examines vulnerability to food insecurity using the Vulnerability as Expected Poverty framework. The dataset used was large and comprehensive to develop robust profiles of vulnerable households. This is executed employing the sustainable livelihoods framework. The findings show that human and financial capital plays a critical role in making rural households resilient from vulnerability to food insecurity. The failure of natural resources to support agricultural livelihoods emerged as an important factor for rural household vulnerability to food insecurity. Gender-based imbalances still prevail, explaining most of the rural household vulnerability to food insecurity. Female-dominated households still endure most of the prevailing vulnerabilities to food insecurity, and this is even worse for households headed by younger females. Policies, strategies, and institutions in South Africa have not been able to address household vulnerability to food insecurity. The study identified Eastern Cape and KwaZulu-Natal as the most vulnerable provinces where food policy has to be a top priority agenda.

## 1. Introduction

Evidence shows that malnutrition and food insecurity rates have risen in most parts of South Africa, although the country is generally known to be nationally food secure [1]. There is the recognition that the affordability, availability, and quality of food remains a challenge, with South Africa ranking 40 out of 105 countries, with a 61% food security aggregate score [2]. Furthermore, there is a need to highlight that any intervention has to be approached with the understanding that food insecurity is a complex and multi-faceted rural development endeavor [3], linked to health and the environment [2], affecting the health, life, and well-being of households [3]. To further compound these food insecurity challenges, the country is also facing high unemployment levels, HIV and AIDS pandemic, and inadequacy of essential service delivery [4], challenges that have been exacerbated by the current COVID-19 epidemic. The quantum of these issues could have cumulative adverse effects on household food (in)security. Urgent actions are required for which empirical evidence is an input [2].

However, these urgent actions should be taken with a clear understanding that food insecurity is not a static concept; current food security does not guarantee future food security [5]. This is where the “forward-looking concept”—vulnerability—becomes imperative. Vulnerability to Food Insecurity (VFI) is a function of not only exposure to shocks but also the capacity of each household to deal with the welfare impacts of the shocks. The level of risk exposure and the capacity to absorb shocks are changing, making vulnerability dynamic. Due to their ability to capture these dynamic qualities of food insecurity, vulnerability assessments are considered more robust, and they hold high development policy interest [6,7]. They can inform targeted interventions on how to protect households from the livelihood impacts of shocks because what really matters for policy purposes is the dynamics.

Moreover, the Sustainable Livelihoods Framework is very critical when executing vulnerability to food insecurity assessments because of its detailed and overarching approach to understanding how people make a living [8]. A sustainable livelihood is defined as capabilities, capital assets (financial, physical, social, human and natural) and activities required for a means of a living which can resist stresses and shocks such as drought and floods [9]. A livelihood is sustainable when it can cope with and recover from stresses and shocks and maintain or enhance its capabilities and assets both now and in the future [10]. Livelihood is, therefore, broader than income and it represents the capabilities available to households to follow different livelihood strategies [9].

“The framework has been seen as a vade-mecum for vulnerability assessment” [11]. It is an instrument for understanding the complexities of household livelihood systems and their interaction with the outside environment [12]. It is firmly centered on five components, namely, vulnerability context, capital endowment, Institutions and policies, livelihood strategies, and livelihood outcomes. Vulnerability context refers to unexpected events that may undermine household asset base and put households at risk of falling below the poverty line; these shocks can either be covariate or idiosyncratic. Access to household assets is influenced by policy and institutions. Livelihood strategies refer to mainly choices with a combination of economic activities that talk to households’ use of these resources and understanding of institutions revolving around them. All these interactions collectively influence the ultimate household livelihood outcomes.

## 2. Problem Statement

This paper is embedded in the theory that households with a strong asset base are more likely to be safe and sustain their per capita consumptions in the event of shocks than households with less capital. The capacity to withstand shocks is defined by decisions (livelihood activities) these households make on the use of these resources and the support they get from well-functioning institutions, which are assumed to effectively implement impactful policies. With these enabling policies and accessible institutions, households sustainably enjoy a broader asset base, wider livelihood options, and reduced vulnerability to shocks. However, taking it from the above-mentioned livelihoods and vulnerability issues still plaguing rural households in South Africa, secure access to food and low vulnerability cannot be guaranteed [2]. Households still face poor access to resources, and the impact of these shocks and households’ resilience will continue to be of interest for research and development [13]. Furthermore, this approach (vulnerability) enables researchers to have a deeper understanding of the exposure and sensitivity households have to livelihood shocks. Variations in household asset ownership and the ability to deploy them productively have been identified as critical factors in locating vulnerable groups [13]. The ability to identify these vulnerability groups is critical because it allows social support to be temporally and spatially targeted, avoiding these groups from sliding to destitution when shocks occur.

Given the complex nature of household vulnerabilities, it is worth noting that vulnerability assessments should ideally be attempted with panel data of length and richness [14]. However, such data are very scarce, especially in the developing world. What is relevant for countries, such as South Africa, is a comprehensive household survey with detailed information on household characteristics, expenditure patterns, and income [5]. Hence, in this study, vulnerability was measured using the “Vulnerability as Expected Poverty” (VEP) model [14]. This model measures household vulnerability to food insecurity by estimating the expected mean and inter-temporal variation in household food consumption using more practical cross-sectional or short panel data [15]. There are already a number of studies on VFI using VEP model (e.g., [7,13,14,15,16,17,18,19,20]). Despite these and many other empirical studies, a number of gaps still exist demanding further research.

The first contribution of this paper is the exhaustive use of SLA framework to measure household vulnerability. While reviewing the above-mentioned VIF studies using VEP, the frequently used indicators are household capital endowments, livelihood strategies, and for shocks, a recall on the number of household members who died/fell ill in a certain period is used as a proxy for idiosyncratic shocks [13]. Even though understanding the livelihood implications of this shock is critical, there are a number of other household vulnerability contexts not accounted for, and they are listed in Table 1 below with illustrative examples. Even though the listed types of household vulnerability are mainly covariate (communal/regional level) and would require models beyond the standard VEP model, this study has identified structural vulnerabilities that can be measured at a household level. Exploring these other dimensions of household vulnerabilities is essential in providing in-depth insights about these issues at the household level and development of references for policy evaluation [12]. While studies such as [6,15,20] have investigated if the nature of the vulnerability is structural or risk induced using a multilevel approach (from a household to different structures of societies), this study aims to understand the nature of these structural vulnerabilities within a household. Moreover, this study also has included a score for a total number of environmental problems experienced by each household, to account for vulnerabilities that may arise as a result of the failure of natural resources to support their livelihoods. Thus, this relationship will be studied, with its policy implications.

The second gap is the estimation of variables. Many indicators of food (in)security and vulnerabilities are reported at a household level [21]. As useful as these indicators may be, heavy reliance on them, however, neglects individual dimensions, which may give more clarity on the underlying factors of household vulnerability to food insecurity [21]. For instance, [22] shows how using only a household head gender tends to underestimate gender differences in agricultural productivity. Ref. [21] shows how focusing only on household-level coping mechanisms may alter our understanding of the heterogeneous impacts of shocks due to age and gender differences. This prevents policymakers from identifying individual differences and obstacles that may bring more insight into household vulnerabilities and enhance policy interest. A hierarchical model, which is beyond the scope of this study, has been recommended to address this estimation problem [22]. In this study, however, instead of giving household head estimates, all demographic variables have been estimated as either proportion, aggregate scores or household averages. This is with the exception of household head age, as it perfectly correlates with household education scores.

The other contribution of this study pertains to the dataset used. As it has been mentioned above, the household vulnerability can be estimated without lengthy panel data. However, other mandatory requirements, such as a large dataset, still stand. The limitation with smaller sample sizes comes from the assumption that present cross-sectional variance can be used to estimate the future inter-temporal variance in food consumption [14]. When smaller sample sizes have been used to measure VFI, inter-temporal variations in household food consumption generated will remain ambiguous. In the context of Africa, studies such as [7,13] have applied the VEP model in cross-sectional datasets with less than 500 sample sizes; [23] was 1000 households. While these studies are very informative and hold a strong methodological rigor, they are less likely to represent the vast households’ socio-economic dynamics accurately. Generalizing these results to a broader population for policy inferences remains ambiguous. This study addresses this gap by applying the VEP model to a sample size of 5520 rural households. This is the first South African study to have modelled household vulnerability using a VEP model on a national dataset.

The remaining sections are organized as follows. Section 3 is data collection and sampling framework, Section 4 is the empirical model, Section 5 breaks down all variables in the VEP model and presents the hypotheses revolving around them, Section 6 is data analysis, and then Section 7 is conclusions and policy recommendations.

## 3. Data Collection and Sampling

The data used for the analysis was extracted from the General Household Survey (GHS) 2018, which is a yearly cross-sectional national study by Statistics SA. The target population of the survey consists of all households in all nine provinces of South Africa. For this study, however, only data for rural areas across the country were analysed. After data cleaning processes, the sample size came to 5520 rural households. Stata (version 15) by StataCorp LLC, TX, USA, was the statistical software programme used for data analysis.

The sample design for the GHS 2018 was based on the 2013 master sample. The master sample used a two-stage, stratified design with a probability-proportional-to-size (PPS) sampling of primary sampling units (PSUs) from within strata and systematic sampling of dwelling units (DUs) from the sampled PSUs. A self-weighting design at the provincial level was employed, and master sample stratification was conducted at two levels. Primary stratification was defined by metropolitan and non-metropolitan geographic area types. During secondary stratification, the Census 2001 data were summarised at PSU level. The following variables were used for secondary stratification: household size, education, occupancy status, gender, industry, and income.

## 4. The Empirical Model: Vulnerability as Expected Poverty

Vulnerability analysis seeks to explain the underlying forces that cause individuals and families to be (un)able to cope with uncertain adverse shocks (e.g., drought, losing a breadwinner, etc.). The severity of being vulnerable to food insecurity depends on the characteristics of the adverse event and how responsive that particular household is to risk, which, in turn, depends on its asset base [24]. A considerable movement of households into and out of food insecurity received increasing recognition and necessitated the focus on household vulnerability as the building block for social protection strategy [23].

Analysis of VFI requires a model that can generate empirical results on the specific determinants of future food insecurity, using cross-section data. One such empirical model is “Vulnerability as Expected Poverty” (VEP). It draws from the methodology proposed by [14], who estimated the expected mean and variance of food consumption expenditure, using cross-section household living standard measurement survey data. The methodology was further elaborated by [25], who provided quantitative tools for undertaking risk and vulnerability assessments.

Vulnerability is the result of the recursive process as current socio-economic characteristics of households, their exposure to risk, and their capacity to absorb the shocks determine their risk-management capacity [26]. Accordingly, the vulnerability of a household to food insecurity at time *t* (*V_ht_*) is defined as the probability that the consumption (*C*) of the household at time *t* + 1 (*C_h_*_,*t*+1_) will fall below the benchmark (minimum daily consumption, *Z*). That is

(1) $$V_{ht}=P_{r}\left(C_{h,t+1}\right)\leq Z$$

where $C_{h,t+1}$ is the per-capita consumption level of the household at time *t* + 1; and *Z* is the minimum threshold. In South Africa, this is measured by the minimum daily consumption required to meet the inflation-adjusted national poverty line of R785/capita/month (in April 2018 prices) [27], which is the cost of a minimum household basket of goods and services that would satisfy the necessary daily requirements of per capita 2261 kilocalories. This means that a household is regarded as vulnerable to food insecurity if its future expected expenditure per capita is predicted to be less than this amount. The expected mean consumption level is determined by household resource endowment, whereas the variance (or volatility) in household consumption is determined by the frequency and severity of idiosyncratic and covariate shocks, as well as the capacity of the household to cope or the strategies adopted to ensure smooth consumption despite the shocks [15]. This approach starts with an empirical derivation of a variant of VEP from the food consumption expenditure function as:

(2) $$\ln C_{h}=\beta X_{h}+\varepsilon_{h}$$

The main hypothesis in using VEP is that the error term (*ε_h_*) explains the inter-temporal variance in consumption, i.e., it captures idiosyncratic shocks that contribute to differences in food consumption patterns of households that share the same characteristics. The variance with the error term is assumed to be explained by livelihood assets and factors enhancing capability, as in Equation (3) below:

(3) $$\sigma_{e,h}^{2}=\theta X_{h}+\pi_{h}$$

For parameters to be consistent, it will remain necessary to allow heteroscedasticity, depending on *X_h_*. One way to account for this is to obtain the estimates of *β* and *θ* using three-step Feasible General Least Squares (FGLS) [28]. Using the estimated $\overset{\wedge }{\beta }$ and $\overset{\wedge }{\theta }$, the expected log food consumption expenditures and the variances thereof can be computed for each household, as in Equations (4) and (5) below:

(4) E[lnCh|Xh]=Xhβ∧

(5) $$E[\ln C_{h}|X_{h}]=\theta X_{h}$$

Let Φ denote the cumulative density function with the assumption that food consumption expenditure is log-normally distributed. Using the estimated parameters, the probability that a household will fall below the minimum food security threshold (${\overset{\wedge }{V}}_{h}$) in the near future (say time *t* + 1) can be estimated as in Equation (6) below:

(6) $${\overset{\wedge }{V}}_{h}=\overset{\wedge }{P}\left(\ln C_{h}<\ln Z/X_{h}\right)=\Phi \left[\frac{\ln Z-\ln{\overset{\wedge }{C}}_{h}}{\sqrt{{\overset{\wedge }{\sigma }}_{h}^{2}}}\right]$$

Equation (6) represents an ex ante vulnerability measure that can be estimated using cross-section data. A value of 0.5 will be used as the cut-off point to distinguish the “vulnerable” from the “non-vulnerable”, following [26].

Finally, there are two indices computed using PCA, namely, “household asset” and “service delivery” index. They were included in the VEP model as explanatory variables. Principal Component Analysis (PCA) is a multivariate statistical technique used to reduce the number of variables into a smaller number of dimensions. It creates uncorrelated indices where each index is a linear weighted combination of the initial variables. PCA can be defined by Equation (7):

(7) $$PC_{m}=\alpha_{m1}X_{1}+\alpha_{m2}X_{2}+\cdots +\alpha_{mn}X_{n}$$

where $\alpha_{mn}$ represents the weight for the *m*th principal component and the *n*th variable. These PC weights are given by eigenvectors of the correlation matrix, and the variance for each PC is given by the eigenvalue of the corresponding eigenvector. As a result, these components are ordered with the first component explaining the majority of the variation in the original data subject to the constraint that the sum of squared weights is equal to one.

In the 2018 GHS, information on 24 household durable assets, which included various household assets (such as TV set, radios, deep freezer, tumble dryer, cell phones, and vehicles) was used to compute the household assets index. The first three PCs accounted for almost 80% of the total variation and were used to predict the index. The service delivery index was constructed based on farmers’ access to agricultural grants and loans, training, advisory services, improved seeds and fertilizer, and veterinary livestock services. The first five PCs, explained about 81% of the total variation in these variables, were retained for computing this index.

Descriptive analysis (means, frequencies, and standard deviations) was used to better inform the selection of the variables for conducting the PCA. Household asset ownership ranges from a widely varied number of cell phones (max = 14) to a less varied number of TV sets (max = 1 TV sets). Even though service delivery indices are constructed using binary variables (subject to secondary data limitations), the nature of these services varies considerably, from inputs to advisory services, from crop to livestock services. Using such a varied number of household assets and government services was meant to ensure that data used is broad enough to avoid clumping of truncation problems.

## 5. Description of the Variables Used to Explain Vulnerability

Instead of modeling vulnerability as a binary choice model, the approach adopted here conceptualizes vulnerability as a continuum taking household food consumption per capita. Despite its data requirements, this model remains the most widely accepted measure of vulnerability to food insecurity [13]. The quantity of household food basket was determined in such a way that a given bundle meets the predetermined level of minimum daily dietary requirements. This basket was valued at local prices, and households were asked the total amount of money they spent (Rands) on food items for the past 30 days. Asking respondents to recall the 30-day household consumption was found to be a realistic approach, especially in the interest of accurately computing household food consumption per capita (response variable).

Summary statistics of the variables hypothesized to influence future household consumption, with their expected signs, are shown in Table 2. The analysis is based on a hypothesized relationship between the explanatory variables and the natural log of household consumption per capita. Starting with human capital under capital endowments, the age of these household heads reveals that, on average, people who are in their earlier 50s are heading rural households in South Africa. These people have grown enough that they are in a better position to establish their livelihoods. This then makes these households less prone to both current and future consumption inadequacies. With household education, instead of looking at the household head level of education, this study uses an average education level across household members. Households with higher education scores imply strong human capital, and these households have better employment opportunities and are more resilient to future economic shocks. Better access to quality basic education enables members to secure remunerative jobs, which will make them less vulnerable to food insecurity [29]. However, the statistics show a lack of education among the sampled households, most being dominated by members with primary education. If these household members are still minor and at their early stages in terms of education, the current level of household food insecurity will be more of a transitory story.

With financial capital, households with more diverse income sources are less vulnerable to food insecurity because they have more comprehensive livelihood options to withstand shocks. Investments and savings play a critical role also during shocks, and they can be used to acquire more capital, which reduces vulnerability. Remittances increase and diversify household income sources and can reduce household vulnerabilities. The larger the amount of money a household receives as gifts or the more the number of people (networks) it can rely on when facing shocks, the less the chances of being vulnerable to food insecurity, ceteris paribus. Enterprise diversification was also included in the model to see if it reduces household vulnerability. This included livestock, poultry, grain, fruits, and vegetable farming enterprises. Farming across these enterprises reduces risk, and households are more likely to have stable incomes, which will enable clear budgeting and less volatile farming business. This promotes steady household food consumption and less vulnerability. That is why a positive relationship is expected.

When moving onto natural capital, households with cultivated land are likely to turn farm produce into income, improving access to food more than households that own idle land [30]. That is why this variable is predicted to have a positive effect, which is also the case for access to irrigation. Irrigation reduces the risk in agricultural production and improves crop yields, especially in a farming system frequently faced with drought. This implies higher on-farm income and stable food consumption patterns [13]. Environmental stress variable refers to the number of environmental challenges households experience, ranging from irregular or no waste removal to littering, water pollution, soil erosion, overgrazing, and deforestation. In contrast to natural shocks such as drought and floods where people have no control, natural resources degradation is mostly man-made. Households located in areas where the natural resources are not in a state to support their livelihoods are expected to experience frequent environmental stresses and be more vulnerable, ceteris paribus.

Ownership of physical capital (such as livestock) was included and is expected to have a positive influence on the future food security status of households [29]. This is because livestock can be used as collateral since acquiring credit and selling livestock are the common strategies that households employ when facing idiosyncratic shocks. The household asset index was also included in the model. A brief description of this index was given in the preceding section. A household with a higher index is expected to be less vulnerable to food insecurity. Regarding social capital, being a member of societal groups (such as stokvels) enhances social networks and connections. These connections play a role when one faces idiosyncratic shocks, and people who are connected have higher job opportunities. Therefore, households with members who are part of these social groups are less vulnerable.

The number of household members who fell ill and those who were injured in the previous three months was included in the model as proxies for idiosyncratic shocks. A larger number of household members who either fell sick or injured implies low household labor productivity and high vulnerability. As for the structural vulnerabilities, instead of adding gender of the household head like studies in the past, in this study, gender is captured as the proportion of women in a household. It is meant to represent the challenges and vulnerabilities endured by households dominated by females. This would also enable one to tell household socio-economic implications that come with an additional female household member. It is shown in Table 2 above that, on average, females constitute about half (51%) of the household members. Households dominated by males are expected to be less prone to food insecurity due to better opportunities for them to access productive assets such as land and financial capital [31]. Females, however, are less likely to access these assets due to limited access and control caused by cultural and institutional hindrances [4]. Due to all kinds of gender biases documented in the literature, households with larger female proportions are expected to have a lower chance of escaping future food consumption deficiencies. The same hypothesis can be made for households with a large proportion of people living with a disability (SHARE_DISABLE), i.e., they are expected to be more vulnerable because of structural obstacles they face.

Social grants are public transfers in cash, aiming to provide income security, food security, better nutrition, and access to essential services [32]. Even though this program has assisted many vulnerable rural households in South Africa, its potential negative impact on nurturing a culture of entitlement and expectations by rural people cannot be ignored. A study by [33], for instance, found that social grants reaching the non-target family members, creating disincentive effects that impede entrepreneurial development in rural farming households. Moreover, given the absence of legal means of enforcing how this money should be spent, the relationship between social grants and household vulnerability is unpredictable. It is upon the onus of each household to ensure that the child support grant is used on the child’s nurturing as the policy intends, which will ultimately contribute to household human capital. Seven variables on government services were used to construct the service index for each household. This variable is expected to be positive because having access to these government programs and interventions should provide new opportunities and improve household socio-economic status.

## 6. Empirical Results and Discussion

### 6.1. Explaining Household Vulnerability to Food Insecurity

The model set to explain the expected mean and variance in households’ consumption was estimated after accounting for heteroscedasticity using a Generalized Least Squares. The results are presented in Table 3. The variance for expected per capita food consumption measures household resilience to covariate shocks such as an increase in food prices, drought etc. The F-statistic of the model is highly significant (*p* < 0.000 with 18; 5501 degrees of freedom), meaning there is a significant relationship between expected household consumption and the explanatory variables. The Variance Inflation Factor (VIF) results (a mean VIF of 1.47) confirm that there is no multi-collinearity. Twelve out of eighteen variables are statistically significant with expected signs.

Starting with human capital, variables AGE and EDUC_SCORE were found to have a positive and significant effect on household future food consumption. As expected, education improves the chance that a household stays food secure, consistent with both economic theory and studies in the past (e.g., [13,17,26,29]). This clearly shows how important asset education is to make people versatile to find jobs outside agriculture and earn a living. This is also confirmed in a recent case study from South Africa [34].

The statistically significant coefficients corresponding to age imply that age reduces vulnerability to household food insecurity. This means that ceteris paribus, as people grow older, they accumulate assets/experience and adopt effective strategies to cope with shocks, reducing their susceptibility to food insecurity. This is further shown by low variance for household future consumption patterns, implying that this knowledge and accumulated assets enable households to form resilience towards vulnerabilities that come with shocks such as drought.

All the financial capital variables, except remittances, were found to have a positive influence on future household food security. This includes households with multiple income sources, which implies high productivity and low dependency amongst household members. The enterprise diversification results also imply that a household with diversified agricultural enterprises and livelihood options can lower risk and achieve stronger resilience. These results coincide with [13]. The results further show the importance for households to have a strong investment/savings base to withstand shocks and achieve low vulnerability. Households with a higher number of people with active investments hold a better possibility of future food security with lower inter-temporal food consumption variations. In contrast to other financial assets, receiving gifts (remittance) that form extra income for households was associated with higher vulnerabilities. This may be due to that gifts are not a secure and sustainable source of income in a way that they may build resilience for these households against future shocks. Moreover, remittances are typically received by older people who have retired, with limited asset base, using the remittances for consumption to sustain the household.

As for the natural capital, the results reveal that households that irrigate their crops face low risk from covariate shocks such as drought, and they tend to have stable consumption patterns than households that do not irrigate. The findings further show that households located in places plagued with environmental stresses face high VFI. These households also face high risk from covariate shocks. When natural resources and environmental amenities deteriorate, it results in limited livelihood options for surrounding rural inhabitants.

Regarding vulnerability context, results show that households with a high number of members who fell sick are highly vulnerable. When it comes to household structural vulnerabilities, the findings show that food consumption vulnerabilities exist in households dominated by females and people living with a disability. After more than two and a half decades of political rhetoric on economic inclusivity and women empowerment, households dominated by males still stand higher chances of escaping vulnerability to food insecurity. People living with disabilities remain marginalized too.

For the institutions, results reveal that households with a large number of social grant recipients are more vulnerable. This is because even though social grants have played a critical role in providing a safety net for poor households, they, however, may entrench a culture of expectations and entitlement. [33], for instance, found that social grant dependency was negatively associated with agricultural entrepreneurship. This implied that social grants had benefited the non-targeted household members, which, in turn, created disincentive effects that inhibit entrepreneurship development. In a study by [35], South African households receiving social grants were found to be more food insecure with lower mean monthly food expenditure, lower dietary diversity, and lower wealth index. The findings further reveal that access to government-related services such as loans and grants fail to combat household vulnerabilities to food insecurity on a sustainable basis. They only provide short-term relief. This reinforces the need to redefine such programs in such a way that they can support rural households to protect themselves against economic shocks and vulnerability.

### 6.2. What Are the Characteristics of Highly Vulnerable Households

Once the vulnerability index (which ranges from zero to one) is ascribed to each household, one can now start to unpack its characteristics to gain a deeper insight into household vulnerabilities. Using a method proposed by [26], households were classified as either being “less vulnerable” or “highly vulnerable” by a cut-off of 0.5. This exercise shows that at least one third (35%) of the sampled households are highly vulnerable to food insecurity, and the remaining face relatively less vulnerable.

Regarding the food security classifications, the food poverty line of R785 (in April 2018 prices) per capita per month was used as a threshold. Households with per capita consumption below the specified poverty line were classified as current food insecure while those equal and above the poverty line were classified as currently food secure.

The prevalence of poverty in South Africa is confirmed in Table 4 below, i.e., about 27% of the sampled rural households are chronically food insecure. In the poverty and vulnerability to poverty literature, chronic poverty is defined as the group that is currently food insecure, highly vulnerable and consumption is expected to remain below the food poverty line. These rural households are trapped in poverty because they are currently food insecure, and they face very slim chances of escaping the situation. The results further show that Eastern Cape and KwaZulu-Natal are provinces with the highest proportions of households living in chronic food insecurity. Households and individuals that are chronically poor or food insecure are likely to experience severe food insecurity in the long term because of their weak livelihoods and assets base [36]. Household resilience was found to be significantly and positively related to future household food security [37]. North West and Gauteng have the lowest vulnerabilities compared to other provinces. The association between current food security status and vulnerability to food insecurity was tested using the Pearson Chi-Squared test. The test was statistically significant at 1%, suggesting that there is strong statistical support for associations between vulnerability and food security statuses as shown in Table 4.

A number of factors have been identified to provide deeper insights into the dimensions of vulnerability to food insecurity.

#### 6.2.1. Gender Dimensions

The results in Table 5 below further reveal the gender imbalance in rural household vulnerability to food insecurity. The differences are statistically significant at 1%, and they reinforce the need for policy reforms to address the imbalance.

It is apparent from Table 5 that male-dominated households have a lower vulnerability. That is why households that are both food secure and low vulnerable are male dominated. On the other hand, the majority of female-dominated households endure chronic food insecurity. The households that are in transitory food insecurity (currently food secure but face high vulnerability) are female dominated.

The results in Table 6 reveal that special focus needs to be on households headed by females less than the age of 35 since these households face a high probability of chronic food insecurity. Even though households headed by females above the age of 65 years face lower vulnerabilities, about 16% of these may be currently food secure. However, this phase is transitory, and they face high vulnerability. For older adults (35–65 years), the results were split between the low vulnerable and those chronically food insecure.

Overall, the empirical evidence suggests that, despite more than 25 years of South Africa’s democratic dispensation, gender-based socio-economic inequities still remain a policy priority and they need to be addressed through gender empowerment interventions in South Africa. It is essential to correct formal and informal institutional hurdles that result in entrenched gender bias.

#### 6.2.2. Social Grant-Related Dimensions

**Figure 1:**
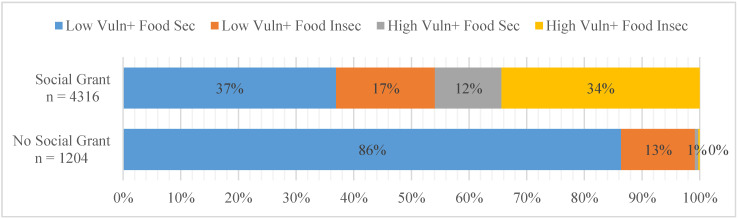
Household vulnerability by whether a household receives a social grant or not (*n* = 5520). Source: Stats SA General Household Survey (2018).

When it comes to social grants, the results in Figure 1 show that about 78% of the sampled households have at least one member who receives one type of social grant. This shows how widespread the coverage of this grant scheme is, how important it is, and how deep rural poverty in South Africa is. The results further show that the majority of social grant non-recipient households (about 86%) had a low vulnerability to food insecurity. Conversely, households receiving social grants showed more signs of susceptibility since about one-third of the recipient households are chronically food insecure.

The findings reported in Table 7 show that about one-fifth of the social grant recipient households receive only Old Age Grant (OAG), almost half receives only Child Support Grant (CSG), and a quarter of them receive both grants. This leaves only 4% to other types of grants. The results show that almost two-thirds of households receiving old age grants are both food secure and face low vulnerability. Meanwhile, chronic food insecurity exists in households receiving child support grants. The different impacts between the OAG and CSG correspond with different sizes of these grants. Households that heavily rely on CSG are more prone to future food insecurity because of the higher dependency ratio.

With household vulnerability that has been shown in the preceding results, the role played by social grants as a safety net scheme in poverty-stricken rural households cannot be ignored. However, what this graph suggests is that more value-adding and productive safety-net programs need to be done for these households to escape vulnerability.

#### 6.2.3. Agricultural-Related Dimensions

The results in Table 8 below show the role played by agriculture in rural households of South Africa is to be the extra source of food, i.e., it serves as a top-up to supplement other sources. However, being the extra source of food proves to be insufficient and unsustainable since the majority of these households are chronically food insecure. These vulnerabilities could be explained by the seasonal variation in access to food, i.e., high food security levels during harvest seasons and chronically food-insecure before the harvest season.

Most households where agriculture is the main source of food were found to be chronically food insecure. The farming activities they are engaged in are not able to feed the family, let alone have a marketable surplus. This reinforces that food (in)security in rural South Africa is by and large a question of access via entitlements. These results show that diversifying their income sources will enable these households to supplement the shortage through purchase, afford other non-food necessities, and build resilience.

## 7. Conclusions

Given the lack of empirical evidence for South Africa, this study examined the prevalence, sources, and distribution of rural household vulnerability to food insecurity. The empirical findings suggest that current food (in)security does not translate to future food (in)security and vice versa. This problem needs to be treated as dynamic, requiring policies and strategies to address not only current food insecurity but also vulnerability. In this sense, vulnerability analysis better informs policy than static food (in)security analysis.

Human and financial capital are found to enable rural households to be more resilient to VFI. This suggests continued support of initiatives aiming to improve access to education in rural areas. The significance of the financial variables suggests the importance of financial literacy, external locus of control—remittances, and diversification of income/enterprise in rural households.

Irrigation proves to be a viable option when facing weather-related shocks. This calls for continued support to irrigating farmers and support for those who may be currently farming dryland and may want to shift to irrigation or diversify the portfolio of their crop enterprises. Farmers need to be informed about good environmental practices and the consequences they will have to endure should they decide to ignore these calls.

Households still lack the necessary capacity to withstand idiosyncratic shocks. Poor household health status exposes rural households to VFI. This reinforces the need for improving health infrastructure, access to nutritious foods, and good hygiene. The adversity of structural vulnerabilities in rural households is also evident, especially for rural households headed by younger females who are more vulnerable. Given that these households are often reliant on CSG, which is not adequate to deal with household vulnerabilities, it calls for a need to revisit the program. Other rural development programs/policy interventions need to be mindful of this and complement this program in a way that these gender-dimensional deprivations are fully dealt with. Rural women should benefit from wider livelihood opportunities.

In sum, this study made the following three specific contributions. Employing the SL framework has made it possible not only to identify and understand intra-household vulnerabilities but also to identify critical assets rural households should have access to as protection shield and the possible influence from policy institutions. Better construct of variables allowed us to have a clear understanding of how gender imbalances remain critical for the prevalence of household vulnerability to food insecurity in rural areas. Using a large sample size not only did make the results robust but also allowed the development of a better profile of these highly vulnerable rural households. The study was able to show that chronic food insecurity exists mainly in rural areas of Eastern Cape and KwaZulu-Natal. For future studies, vulnerability differences between rural and urban areas are still of policy interest. Understanding the nature of both covariate and idiosyncratic shocks facing rural households and the role played by different institutions in protecting these households using appropriate models also need a deeper investigation [13].

## Figures and Tables

**Table 1:** Five Components of Sustainable Livelihood Approach Framework.

Sustainable Livelihood Framework	Illustrative Examples	
Types of Household Vulnerabilities	Natural and weather-related shocks	Drought, floods, heatwaves
	Pest and disease epidemics	Disease pandemics, insect attacks,
	Economic shocks	Financial crisis
	Civil strife	Wars, armed conflicts, displacement
	Seasonal stresses	Seasonal rainfall shortages
	Environmental stresses	Land degradation, pollution, bush fires
	Idiosyncratic shocks	Illness or death in the household, shortage of income due to job loss, theft, injuries
	Structural vulnerability	Lack of voice or power to make claims; cultural, gender, age-related hindrances
Types of Capital Endowments	Human capital	Education, health status, dependency-ratio
	Physical capital	Livestock, vehicles, property
	Natural capital	Land, water, grazing, forests
	Financial capital	Savings, sources of income, credit
	Social capital	Kin networks, connections, influence
Types of Institutions	Formal membership organizations	Cooperatives
	Informal organizations	Societal stokvel membership
	Political institutions	Political parties, parliament
	Economic institutions	Markets, banks, private companies
	Socio-cultural institutions	Religion, traditional authorities
Types of Livelihood Strategies	Positive livelihood sources	On/off-farm income, social grants
	Negative livelihood strategies	Poor livelihood strategies due to limited access to resources/disabling policies
Types of Livelihood Outcomes	Positive livelihood outcomes	±income security, ±vulnerability, ±nutrition, etc.
	Negative livelihood outcomes	

Source: Constructed by the Authors based on FAO (2005). URL: www.fao.org/3/a0273e/a0273e04.htm.

**Table 2:** Definition of variables, summary description and expected signs.

Variable Code	Description	Mean (Std Error)	Min (Max)	Expected Sign
lnCons Capita	Natural log of household consumption per capita (Rands)	6.90 (1.08)	4.33 (10.59)	Response Variable
**CAPITAL ENDOWMENT**				
**Human Capital**				
AGE	Age of household head (Years)	53 (16.74)	12 (108)	+
EDUC_SCORE	Average household education in years (where PhD = 27 years)	7 (3.87)	0 (27)	+
**Financial Capital**				
SOURCE_INCOME	Household income diversification (number)	2 (0.69)	1 (5)	+
REMMITANCES	Natural log of amount of remittance received (Rands)	1.33 (2.76)	0 (9.47)	+
HHINV_SCORES	People with active investment accounts in a household (number)	0.48 (0.88)	0 (9)	+
ENT_DIV	Household agricultural enterprise diversification (number)	1.01 (1.28)	0 (4)	+
**Natural Capital**				
IRRIGATION	Access to irrigation (1 = Yes; No = 0)	0.14 (0.34)	0 (1)	+
CULTIV_LAND	Size of land under agricultural production (hectares)	0.23 (0.56)	0 (7.5)	+
ENVIRO_STRESSES	Environmental problems experienced by the household in the last 12 months (number)	3.5 (1.39)	0 (7)	−
**Physical Capital**				
TLU	Livestock holding in Tropical Livestock Units (1TLU = 250 kg live weight of livestock)	0.99 (3.3)	0 (54.4)	+
HHASSET_INDEX *	Index representing asset endowment (movable) per household	0.09 (1.2)	−2.36 (8.25)	+
**Social Capital**				
SOC_MEMBER	Members of social groups per household (number)	0.50 (0.75)	0 (6)	+
**HOUSEHOLD VULNERABILITY**				
NUM_INJURIES	Household members injured in the last three months (number)	0.006 (0.77)	0 (2)	−
NUM_ILL	Household members who fell ill in the past three months (number)	0.34 (0.86)	0 (12)	−
SHARE_GENDER	Female representation in a household (proportion)	0.51 (0.30)	0 (1)	−
SHARE_DISABLE	Share of household members living with disability in the household (proportion)	0.13 (0.25)	0 (1)	−
**INSTITUTIONS**				
SOCIAL_GRANTS	Household members receiving social grants (number)	2 (2.02)	0 (14)	±
SERVICE DELIVERY INDEX *	Household index for agriculture-related assistance from the government	3 (2.24)	0 (14)	+

Source: Stats SA (General Household Survey) (2018). URL: https://www.datafirst.uct.ac.za/dataportal/index.php/catalog/801; * Indices were computed using Principal Component Analysis.

**Table 3:** Coefficient estimates to explain household future mean consumption and its inter-temporal variations.

Variable Name	Log Future Food Consumption Expenditure	Variance of Future Food Consumption Expenditure
	Coefficient (Robust std. Error)	Coefficient (Robust std. Error)
AGE	0.012 (0.001) ***	−0.001 (0.001) **
EDUC_SCORE	0.078 (0.004) ***	0.008 (0.004) *
SOURCE_INCOME	0.565 (0.020) ***	0.129 (0.025) ***
REMITTANCES	−0.036 (0.004) ***	0.012 (0.008)
HHINV_SCORES	0.079 (0.013) ***	−0.026 (0.015) *
ENT_DIV	0.428 (0.047) ***	0.057 (0.176)
IRRIGATION	−0.021 (0.031)	−0.077 (0.040) *
CULTI_LAND	−0.005 (0.025)	0.082 (0.035) **
ENVIRO_STRESSES	−0.043 (0.010) ***	0.024 (0.014) *
TLU	−0.003 (0.003)	0.003 (0.004)
HHASSET_INDEX	−0.004 (0.007)	−0.001 (0.009)
SOC_MEMBER	0.006 (0.014)	0.036 (0.021) *
NUM_INJURIES	−0.053 (0.161)	0.148 (0.012)
NUM_ILL	−0.044 (0.012) ***	−0.016 (0.015)
SHARE_GENDER	−0.330 (0.038) ***	−0.126 (0.068) *
SHARE_DISABLE	−0.057 (0.010) ***	0.002 (0.017)
SOCIAL_GRANTS	−0.114 (0.007) ***	−0.023 (0.009) ***
SERVICE DELIVERY INDEX	−0.015 (0.007) ***	−0.022 (0.007) ***
Constant	5.83 (0.064)	0.629 (0.075)
Number of observations	5520	5520
F (18; 5501)	239	10.90
Prob > F	0.000	0.000
R-Squared	0.47	0.04
Root MSE	0.747	0.731

Source: Stats SA General Household Survey Dataset (2018). URL: https://www.datafirst.uct.ac.za/dataportal/index.php/catalog/801. *, **, ***: Statistically significant at 10%, 5% and 1% respectively.

**Table 4:** Household Vulnerability and Food Security across Provinces (*n* = 5520).

	EC	NC	FS	KZN	NW	GP	MP	LP	%	Total
Low Vulnerability + Food Security	39%	58%	55%	43%	68%	61%	51%	47%	48%	2634
Low Vulnerability + Food Insecurity	18%	14%	12%	17%	9%	15%	18%	17%	16%	894
High Vulnerability + Food Security	12%	7%	7%	9%	6%	6%	8%	10%	9%	506
High Vulnerability + Food Insecurity	32%	22%	26%	31%	17%	19%	24%	26%	27%	1486
%	100%	100%	100%	100%	100%	100%	100%	100%	100%	
Total	1188	168	103	1185	558	54	785	1479		

Source: Stats SA General Household Survey (2018). EC = Eastern Cape; NC = Northern Cape; FS = Free State; KZN = KwaZulu-Natal; NW = North West; GP = Gauteng Province, MP = Mpumalanga Province; LP = Limpopo Province.

**Table 5:** Vulnerability by household gender representation (*n* = 5520).

Household Vulnerabilities		Male-Dominated	Gender-Balanced	Female-Dominated	Overall
Low Vulnerability	Food secure	45%	21%	34%	2634
	Food insecure	39%	18%	44%	894
High Vulnerability	Food secure	22%	21%	57%	506
	Food Insecure	25%	14%	60%	1486
Overall		2016	1037	2467	5520

Source: Stats SA (General Household Survey) (2018).

**Table 6:** Vulnerability and gender of heads of households (*n* = 5520).

Household Vulnerabilities		Female-Headed ≤25 Years	Female-Headed 26–35 Years	Female-Headed 35–65 Years	Female-Headed >65 Years	Male-Headed	Total
Low Vuln	Food secure	17%	30%	35%	42%	64%	2634
	Food insecure	3%	8%	20%	18%	15%	894
High Vuln	Food secure	9%	6%	7%	16%	8%	506
	Food Insecure	70%	56%	37%	24%	15%	1486
		100%	100%	100%	100%	100%	
Total		98	339	1718	879	2436	5520

Source: Stats SA (General Household Survey) (2018).

**Table 7:** Household vulnerability by type of social grant (*n* = 4158).

Household Vulnerabilities		Old Age Grant	Child Support Grant	Both
Low Vulnerability	Food secure	65%	29%	26%
	Food insecure	11%	20%	17%
High Vulnerability	Food secure	22%	5%	14%
	Food insecure	2%	45%	42%
		100%	100%	100%
Overall		874	2130	1154

Source: Stats SA General Household Survey (2018).

**Table 8:** Household vulnerability to food security versus household engagement in agriculture.

Household Vulnerabilities		Main Food	Main Income	Extra Income	Extra Food	Leisure	Unspecified	Overall
Low Vulnerability	Food Secure	57	21	81	735	54	1186	2634
	Food Insecure	21	9	43	338	22	461	894
High Vulnerability	Food Secure	21	2	10	254	23	196	506
	Food Insecure	84	15	32	681	43	631	1486
Overall		184	47	166	2008	142	2974	5520

Source: Stats SA General Household Survey (2018).

## Data Availability

Publicly available datasets were analyzed in this study. This data can be found here: http://www.fao.org/3/a0273e/a0273e04.htm; https://www.datafirst.uct.ac.za/dataportal/index.php/catalog/801.
